# Sonosensitizer nanoplatform-mediated sonodynamic therapy induced immunogenic cell death and tumor immune microenvironment variation

**DOI:** 10.1080/10717544.2022.2058653

**Published:** 2022-04-08

**Authors:** Jing Zheng, Yixuan Sun, Tengfei Long, Dong Yuan, Song Yue, Ni Zhang, Zhu Yang

**Affiliations:** a Department of Gynecology and Obstetrics, the Second Affiliated Hospital of Chongqing Medical University, Chongqing, China; b Department of Oncology, the Second Affiliated Hospital of Chongqing Medical University, Chongqing, China

**Keywords:** Epithelial ovarian cancer, sonodynamic therapy, immunogenic cell death, ER stress, tumor microenvironment

## Abstract

Epithelial ovarian cancer (EOC) is one of the most lethal gynecologic malignancies, and effective treatments are still lacking due to drug tolerance and tumor recurrence. In this study, we aimed to investigate the effects of sonodynamic therapy (SDT) on ovarian cancer and its potential mechanism. Folate receptor-targeted and ultrasound-responsive nanoparticles (NPs) were constructed using PLGA-PEG-FA (PLGA: poly (lactic-co-glycolic) acid, polyethylene glycol (PEG), FA: folate), the reactive oxygen species (ROS)-generating sonosensitizer IR780 and the oxygen-carrying material perfluorohexane (PFH), termed IRO@FA NPs. The antitumor effect of NPs triggered by ultrasound (US) was measured by an apoptosis assay in a C57/BL6 mouse model. Immunochemistry and flow cytometry were used to detect the proportion of CD3^+^ T, CD4^+^ T, CD8^+^ T cells and activated dendritic cells (DCs) in spleens and tumor tissues to assess variation in the immune response. Moreover, endoplasmic reticulum (ER) stress and immunogenic cell death (ICD) markers (high mobility group protein box-1, ATP and calreticulin) were detected to identify potential mechanisms. The results showed that IRO@FA NP-mediated SDT promoted ID8 cell apoptosis both in vitro and in vivo. The densities of CD3^+^ and CD8^+^ T lymphocytes and inflammatory markers were upregulated in tumor tissues. IRO@FA NP-mediated SDT prompted DC maturation and T lymphocyte infiltration by inducing ID8 cell ICD.

## Introduction

Epithelial ovarian cancer (EOC) is one of the most malignant diseases of the female reproductive system; due to its asymptomatic nature at earlier stages, patients are not usually diagnosed until advanced stages (Lheureux et al., 2019). The current therapeutic strategy for EOC is cytoreductive surgery and chemotherapy (Tew et al., 2020); however, patients in advanced stages have a 5-year survival rate of less than 30% owing to systemic toxicity and drug tolerance (Torre et al., 2018; Lheureux et al., 2019). Due to these problems, it is imperative to investigate alternative treatment strategies for EOC.

Noninvasive treatments such as photodynamic therapy are currently applied in clinical settings and have achieved therapeutic effects; however, due to the limited tissue penetration of light, deep-seated tumors cannot be eliminated completely (Abrahamse & Hamblin, 2016; Kwiatkowski et al., 2018). Ultrasound (US)-triggered sonodynamic therapy (SDT) is an ideal alternative method that can achieve deeper tissue penetration without damaging the surrounding healthy tissues (Liang et al., 2020; Son et al., 2020). To perform effective SDT, a good sonosensitizer is important.

IR780 is a multifunctional molecule, and collective data have demonstrated its good reactive oxygen species (ROS) yield. However, IR780 has inherently poor water solubility and direct toxicity when intravenously administered (Alves et al., 2018; Leitao et al., 2020). To overcome the low water solubility of IR780 and improve its biocompatibility, polylactic-co-glycolic acid (PLGA), which has been approved for clinical use by the FDA (Jain, 2000), was used to encapsulate IR780 by a two-step method. It is estimated that most ovarian cancers overexpress folate receptor α (Scaranti et al., 2020; AOCS Study Group, 2014), and folic acid (FA) can be attached to PLGA to provide targeting ability. The therapeutic effects of SDT mainly rely on ROS, acoustic energy-triggered sonosensitizers and surrounding oxygen to generate ROS; because of these features, the oxygen content in the environment plays a decisive role in the therapeutic effect. Hypoxia resulting from abnormal blood vessels and oxygen consumption is a widely recognized pathological feature of the tumor microenvironment (TME) and contributes to poor outcomes (Salmon et al., 2019; Wang et al., 2021). To alleviate tumor hypoxia and improve ROS production by SDT, perfluorohexane (PFH) (Cheng et al., 2015), which has good biocompatibility and oxygen carrying ability, was introduced as a component of the sonosensitizer. Finally, we constructed complex sonosensitizer nanoparticles, with PFH cores surrounded by IR780 and wrapped by PLGA-PEG-FA (PLGA: poly (lactic-co-glycolic) acid, polyethylene glycol (PEG), FA: folate) and termed them IRO@FA nanoparticles (NPs).

The newly generated NPs were demonstrated to have the ability to produce ROS after irradiation with US. An in vivo study showed that IRO@FA NP-mediated SDT exerted antitumor effects and modulated the tumor immune microenvironment. Further study revealed that IRO@FA NP-mediated SDT activated antitumor immunity by inducing ID8 cell immunogenic cell death (ICD) and secretion of damage-associated patterns (DAMPs).

## Materials and methods

### Synthesis and characterization of IRO@FA NPs

Two milligrams IR780 (Sigma–Aldrich, USA) and 50 mg PLGA (50:50, MW: 20,000 Da)-PEG (MW: 2,000 Da)-FA (purity: 91%, Ruixi BIO Engineer Co., Ltd., China) were dissolved in 2 ml dichloromethane (DCM), and then 200 μl PFH (Strem, USA) was added to the mixture, which was then sonicated (Sonics & Materials, Inc. USA) with 65 W amplification for 4 minutes and then for another 3 minutes with 8 mL 4% poly (vinyl alcohol) (PVA). The solution was stirred and evaporated at room temperature for 4 hours. Then, the sample was centrifuged at 8000 rpm/min and washed with distilled water. Finally, the sediment was collected and stored at 4 °C. IRO@FA NPs were diluted in phosphate-buffered saline (PBS), and one drop was placed on a copper mesh and allowed to dry naturally. The next day, the NP morphologic characteristics were observed by transmission electron microscopy (TEM, h-7500; Hitachi). A dynamic light scattering (DLS) analyzer (Malvern Instruments, Nano ZS90, UK)) was used to detect the size distribution of IRO@FA NPs in PBS. The absorption spectrum of free IRO@FA NPs and the encapsulation efficiency were determined by UV–Vis spectroscopy (UV-2600, Shimadzu, Japan) based on a standard concentration curve of free IR780.

IR780 encapsulation efficiency (%) = weight of loaded IR780/weight of total IR780 × 100%

### SOSG assay and *in vitro* IR780 release

ROS production was measured with a singlet oxygen sensor green (SOSG; Thermo Fisher, S36002) assay. NPs distributed evenly in PBS at 5 μg/ml IR780 and SOSG were dissolved in methyl alcohol, and the concentration was adjusted to 10 μM/ml. Then, the solutions were stimulated by US (2 W, 650 kHz), and a fluorescence spectrophotometer (RF-5301PC, Shimadzu, Japan) was used to calculate the fluorescence intensity at 525 nm.

The IR780 release profile from IRO@FA NPs in PBS (PH 7.4) was detected. NPs were distributed in PBS and treated with or without US (2 W/cm^2^, 30S) at 37 °C. Then, the samples were centrifuged at 8000 rpm/min at different time points, and the amount of IR780 in the supernatant was detected by a UV–Vis spectrometer.

### Cell lines and culture

ID8 ovarian cancer cells from C57/BL6 mice were provided by Dr. Luo, Xiaoxiao of Army Medical University, and the cells were cultured in Dulbecco's modified Eagle’s medium/F12 (Gibco, Invitrogen, USA) containing 10% fetal bovine serum and 1% penicillin–streptomycin. ID8 cells were maintained at 37 °C in a humidified incubator with 5% CO_2_.

### *In vitro* toxicity assay and cell uptake

NPs were labeled with DiI (DiIC18(3)) and incubated with ID8 cells for 4 hours, and the rate of the binding of the cells and NPs was calculated by flow cytometry assays. To further determine the cellular distribution of NPs, the cells were seeded in 12-well chambers with coverslips. The next day, fresh medium containing 5 μg/ml IR780 was used to replace the old medium. After 4 hours of incubation, the coverslips were fixed with 4% paraformaldehyde solution, and then the cell nuclei were stained with 4′,6-diamidino-2-phenylindole (DAPI) for 10 minutes. Finally, a laser confocal microscope (LSCM) was used to detect the cellular uptake of NPs. Cells were cultured in 96-well plates, and 24 hours later, the medium was changed to fresh medium containing different concentrations of IRO@FA NPs. After incubation for 4 h, the cells were treated with US (2 W, 30 S), and 24 hours later, cell viability was detected by a CCK-8 detection kit. Finally, the absorbance at 450 nm was assessed by a multimode microplate reader. The antitumor effect was further evaluated by flow cytometry and Calcein AM (green, live cells) and PI (red, dead cells) costaining.

### Measurement of intracellular ROS levels

Cells were cultured as stated above, and their intracellular ROS levels were measured by 2′-7′dichlorodihydrofluorescein diacetate (DCFH-DA; Beyotime Biotechnology, China). In brief, ID8 cells were treated with IRO@FA NPs. Three and a half hours later, the cells were incubated with 10 μM DCFH-DA for 30 min at 37 °C, and excess DCFH-DA was removed by washing with PBS three times. Then, the cells were treated with US (2 W, 30 S) and incubated for another 30 minutes. Finally, the fluorescence signals were viewed by LSCM.

### Mouse model

Six- to eight-week-old female C57/BL6 mice were subcutaneously injected with 3 × 10^6^ ID8 cells in the left flank. Tumor size was measured every 4 days with a caliper using the formula: 0.5 × width^2^ × length. When the tumor volume reached more than 30 mm^3^, the mice were randomly grouped and injected with IRO@FA NP solution (IR780, 1 μg/g) through the tail vein. Beginning 2 h later, the tumors were treated with US (2 w, 300 s) (Zhang et al., 2020) every 4 days. The mouse body weight and tumor volume were recorded. All experiments were approved by the Institutional Animal Protection and Utilization Committee of Chongqing Medical University. Experimental procedures were performed in conformity with the guidelines of the National Institutes of Health Guide for the Care and Use of Laboratory Animals (NIH publication No. 85–23, revised 1996).

### Western blot assay

Cells were homogenized in lysis buffer, sonicated at 4 °C, and then centrifuged at 13000 rpm/min for 15 minutes. The supernatant was collected, and the protein concentrations were detected with a bicinchoninic acid kit. Then, the supernatant was mixed with 4X SDS loading buffer and heated at 100 °C for 10 minutes. Protein (30 μg per lane) was loaded on 10% SDS–PAGE gels, separated, and then transferred to a 0.22 μm polyvinylidene difluoride membrane (PVDF; Millipore, IPVH00010). Then, the blots were blocked with 5% nonfat milk for 2 hours at room temperature and incubated with antibodies against the following proteins overnight at 4 °C: GAPDH, IRE1α, phosphorylated IRE1α (P-IRE1α), CHOP (Cell Signaling Technology, USA), eIF2α, and phosphorylated eIF2α (P-eIF2α) (Abcam, UK). After that, the membranes were washed with TBST and incubated with the corresponding secondary antibodies at 37 °C for 1.5 hours. Then, a chemiluminescence imaging system (Bio–Rad, USA) was used to visualize the protein bands.

### Primary dendritic cell (DC) extraction and culture

Female C57/BL6 mice were euthanized and immersed in 75% ethyl alcohol for 5 minutes. Their femur cavities were flushed with PBS with a syringe and a needle. The fluids were collected into sterile tubes and centrifuged at 1200 rpm for 5 minutes. Then, the supernatant was discarded, and the sediment was resuspended in red cell lysis buffer. Three minutes later, the samples were centrifuged again. Finally, the DCs were resuspended in 1640 culture medium containing 10% fetal bovine serum, 10 ng/ml IL-4 and 10 ng/ml GM-CSF (Absin, China). Every 3 days, half of the culture medium was replaced with fresh medium. Supernatants from the differently treated ID8 cells were collected and added to the DCs. The next day, all DCs were collected and incubated with CD11c or CD80/CD86 antibody (Proteintech, China) in a dark room, and then the proportion of double-positive cells was counted by flow cytometry.

### Immunofluorescence analysis

Cells were seeded on coverslips and treated as described. The coverslips were fixed with 4% paraformaldehyde, blocked with 5% fetal bovine serum (FBS) for 1 h, and then incubated with calreticulin (CRT) at 4 °C overnight. The next day, the coverslips were incubated with the corresponding secondary antibody, and nuclei were stained with DAPI for 10 minutes. Finally, the fluorescence distribution and intensity were observed and analyzed by LSCM.

### Immunohistochemistry

The tumor tissues were embedded and cut into sections. The densities of CD3^+^, CD8^+^, and PD-L1+ cells and the expression levels of high mobility group protein box-1 (HMGB1) and CRT were assessed by the manufacturer’s instructions (Servicebio, Wuhan, China).

#### Elisa

Tumor tissues were homogenized with lysis buffer, sonicated at 4 °C, and then centrifuged at 13000 rpm/min for 15 minutes. The supernatant was collected, and the protein concentrations were detected with a bicinchoninic acid kit. Cell supernatant and mouse blood serum were collected. Then, the levels of IL-6, IFN-γ, and TNF-α in tumor tissues, cell supernatants and blood sera were detected according to the manufacturer’s instructions (Proteintech, China).

### ATP assay

ATP kits were purchased from Beyotime Biotechnology, and residual ATP amounts were detected following the manufacturer’s instructions.

### Transmission electron microscopy

Cells were treated with different conditions, collected and fixed with glutaraldehyde. The structures of the cells were observed under an H-7500 transmission electron microscope (Hitachi, Tokyo, Japan) operated at 100 kV.

### Statistical analysis

The data were analyzed with one-way analysis of variance (ANOVA), and Student’s *t* tests were performed to compare data between two groups. The data are displayed as the mean ± SEM, and *P* < .05 was considered significant.

## Results

### Characteristics of IRO@FA NPs

**Figure 1.**
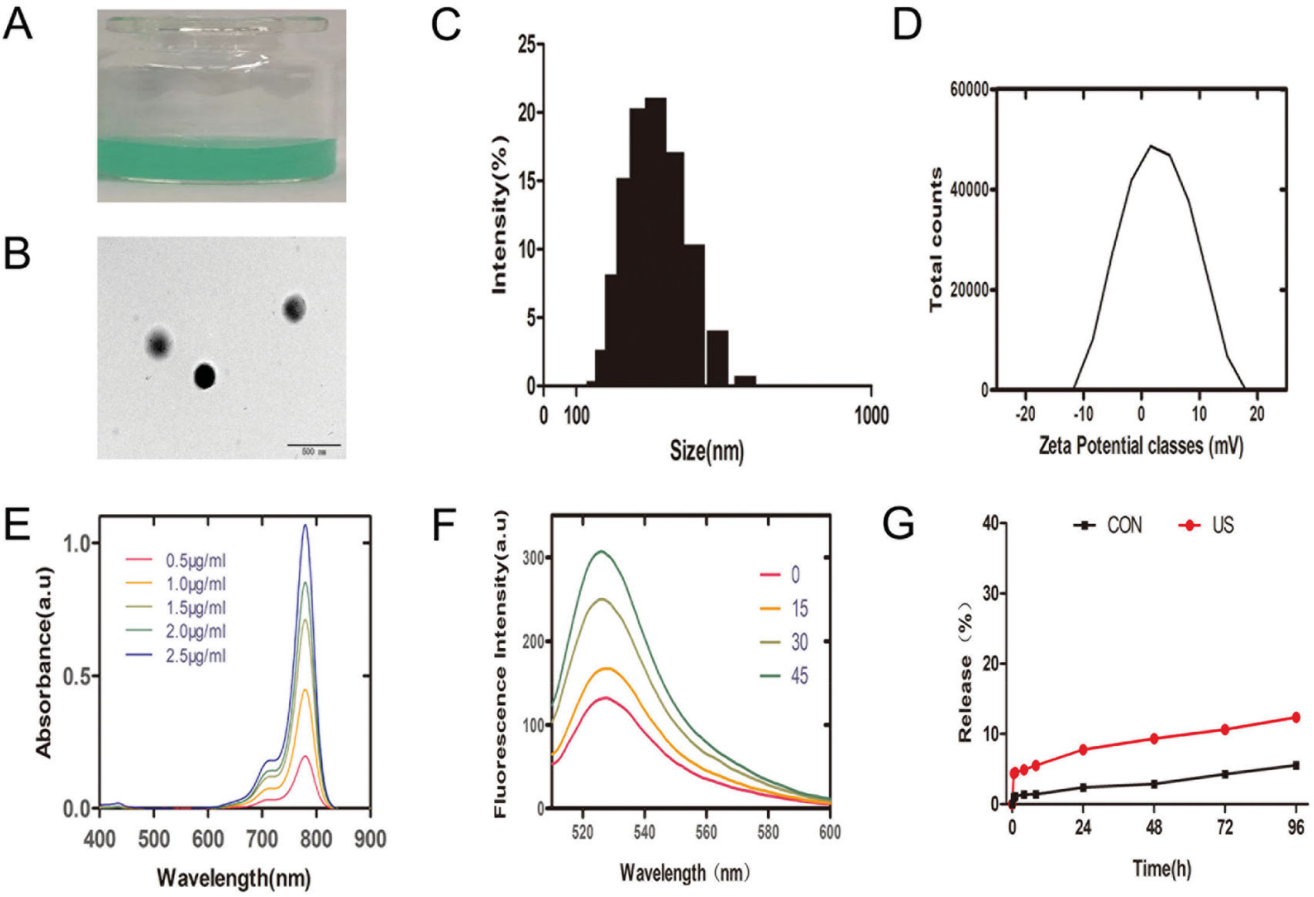
Characteristics of IRO@FA NPs. (A) photograph of IRO@FA NPs in distilled water; (B)TEM image of IRO@FA NPs; (C) size distribution and zeta potential (D) of IRO@FA NPs; (E) UV–Vis absorbance spectra of free IR780 in DCM; (F) Time-dependent SOSG spectra of IRO@FA NPs (5 μg/ml) after irradiated by US; (G) In vitro collective release of IR780 from IRO@FA NPs in PBS.

IRO@FA NPs were synthesized via a two-step emulsion method. The NPs were freely and evenly distributed in distilled water, and the color of the NP solution was dark green (Figure 1(A)). Then, the NPs were diluted with PBS and observed by TEM. The IRO@FA NPs displayed a well-defined spherical shape and were homogenous in size (Figure 1(B)). In addition, the IRO@FA NPs exhibited an average hydration diameter of 313.8 ± 3.2 nm (Figure 1(C)). The zeta potential of the IRO@FA NPs was −1.8 ± 0.4 mV in PBS (Figure 1(D)), and the appropriate diameter of the IRO@FA NPs and their negatively charged surface enabled the NPs to circulate in blood and be taken up by tumors. Free IR780 was dissolved in DCM, and a UV–Vis spectrophotometer was used to detect the UV–Vis absorption spectra of different concentrations and then to draw a standard curve (Figure 1(E)). According to the standard curve and the observed relative absorption value, the encapsulation efficiency of IR780 was 81.9 ± 4.8%.

An SOSG assay was applied to explore the production of ROS. In the SOSG assay, the fluorescence intensity at 525 nm indicated the relative amount of ROS. The intensity of the fluorescence increased with increasing US stimulation time (Figure 1(F)). The SOSG assay indicated that IRO@FA could be a candidate for SDT.

We further investigated the in vitro release of IR780 from the NPs. IR780 release showed similar kinetic curves in the control group and US-treated group, and there was a quick release in the first 24 hours (Figure 1(G)) due to surface-bound IR780 (Liu et al., 2019); in the next 3 days, we observed a slow release curve of IR780, which may be because the hydrophobicity of IR780 makes it difficult to escape from PLGA (Jiang et al., 2015), and a lower amount of IR780 released is also optimal for biosafety.

### IRO@FA NPs were internalized by ID8 cells and produced intracellular ROS

**Figure 2.**
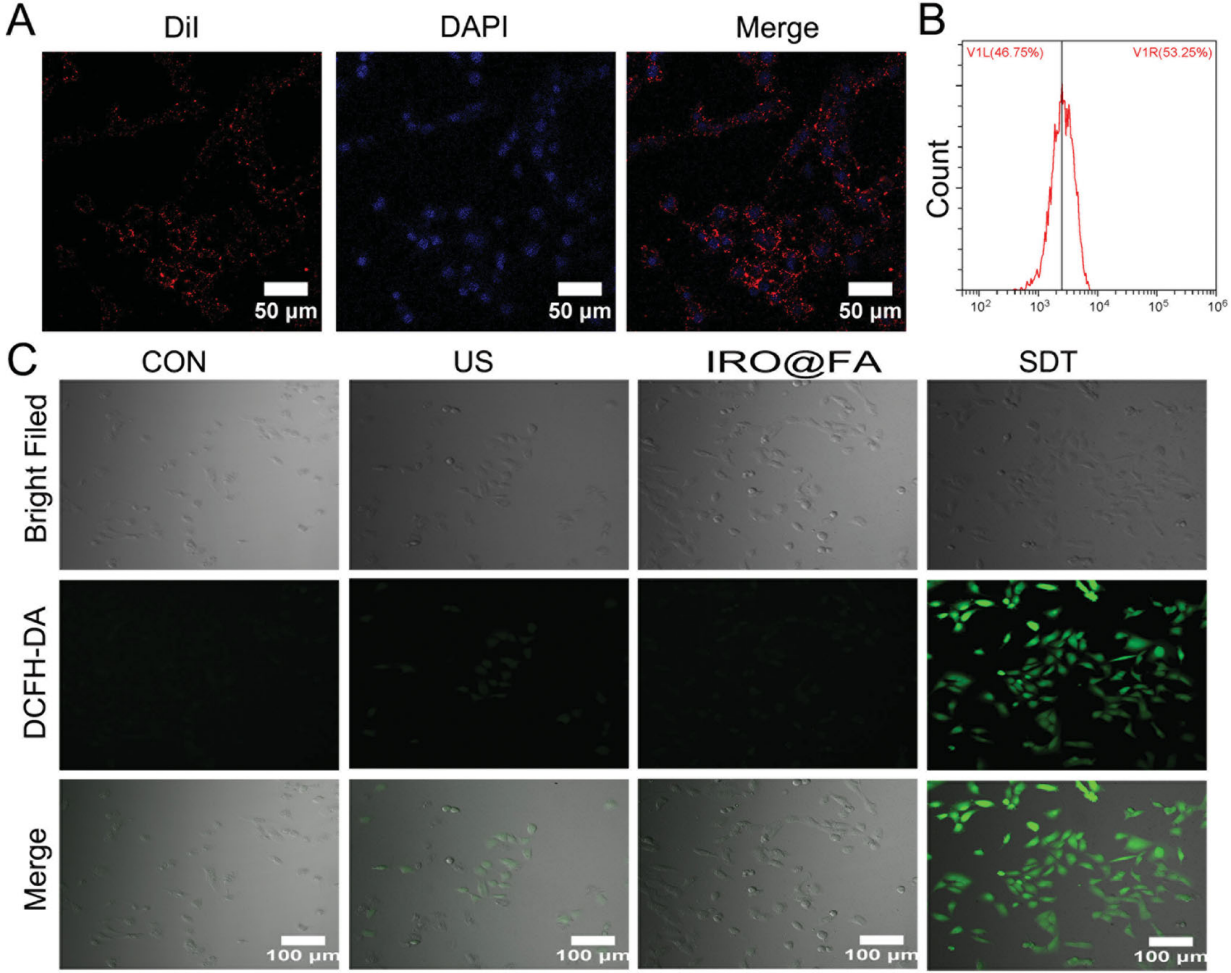
Intracellular uptake of IRO@FA NPs and ROS generation. (A) DiI labeled IRO@FA NPs incubated with ID8 cells for 4 hours observed by LSCM and (B) DiI-positive cells were counted by flow cytometry; (C) intracellular ROS generation was detected with DCFH-DA and observed by LSCM.

IRO@FA NPs were labeled with DiI and then incubated with ID8 cells for 4 hours and imaged with LSCM. Figure 2(A) shows the red fluorescence of IRO@FA NPs internalized into ID8 cells. The cells were collected, and the DiI-positive cells were counted by flow cytometry. Figure 2(B) shows that over 50% of the cells were DiI positive, further demonstrating the good affinity of IRO@FA NPs to tumor cells.

To evaluate ROS production of IRO@FA in vitro, DCFH-DA was applied, and the intensity of fluorescence was observed by LSCM. Figure 2(C) indicates that the fluorescence intensity in the SDT (IRO@FA + US) group was significantly stronger than the levels of fluorescence observed in the other groups. This result indicated that IRO@FA NPs could generate ROS after triggering by US.

### IRO@FA NPs mediated SDT cytotoxicity *in vitro*

**Figure 3.**
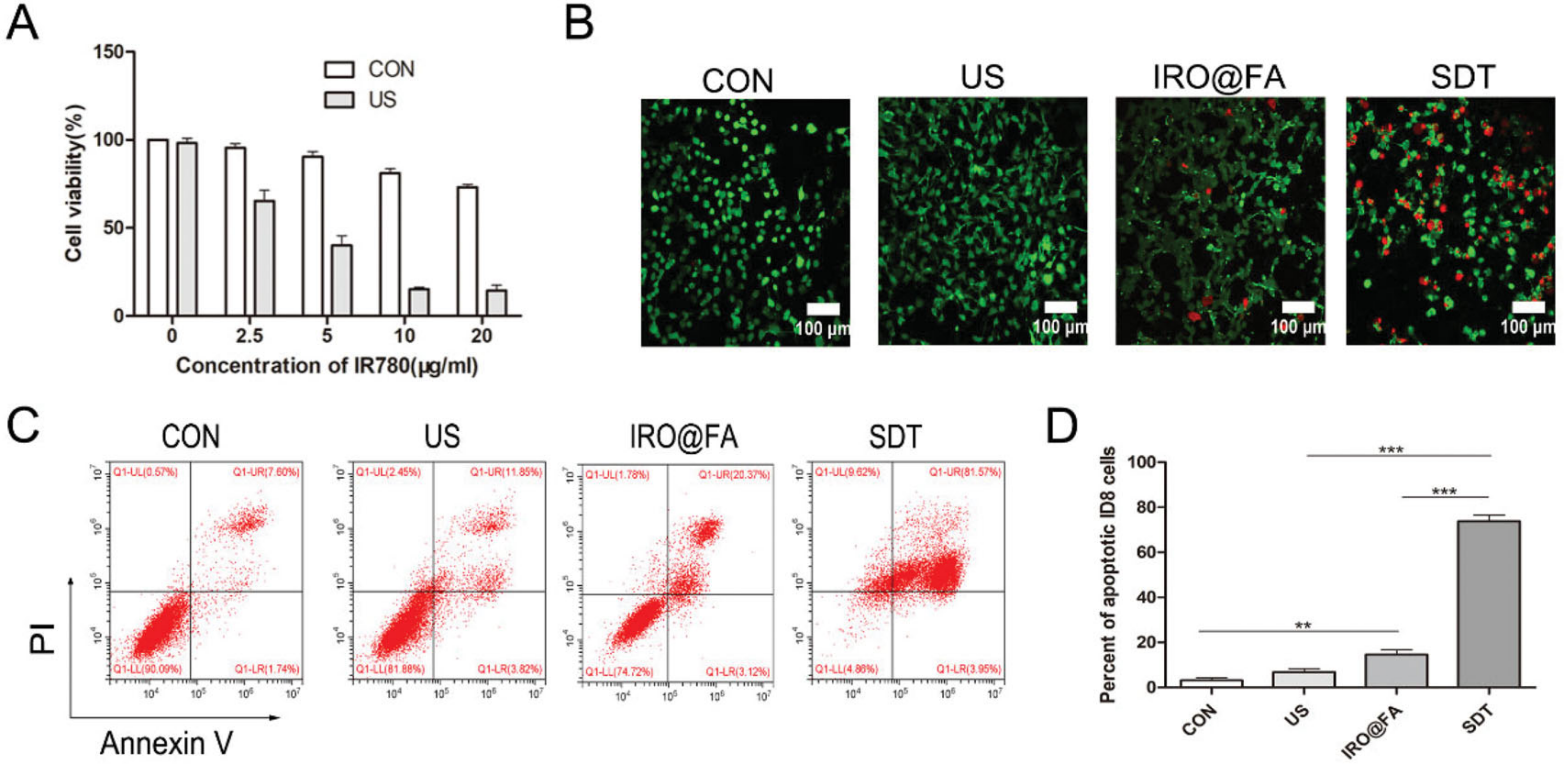
Antitumor effect of IRO@FA NPs mediated SDT in vitro. (A) cell viability after treated with different concentration of NPs; (B) ID8 cells were treated with IRO@FA NPs (5 μg/ml) and US (2w, 30S), costaining of alive (green) and dead cells (red); (C) The proportion of apoptotic cells was analyzed using ANXA5-FITC-PI by flow cytometry and quantitative analysis were showed (D); *p < .05; **p < .01; ***p < .001.

To investigate the antitumor effect induced by IRO@FA NPs and US in vitro, the cytotoxicity of the IRO@FA NPs was assessed with a CCK8 kit. ID8 cells were treated with different concentrations of NPs (0–20 μg/mL) with or without US, and their cell viability was detected (Figure 3(A)). The results showed that IRO@FA NPs or US individually displayed low cytotoxicity in ID8 cells. However, cytotoxicity was significantly increased in the SDT group, indicating the excellent antitumor effect of IRO@FA NP-mediated SDT. The antitumor effect was further evaluated by Calcein AM (green, live cells) and PI (red, dead cells) costaining. The results confirmed that US or IRO@FA NPs alone had little effect on cell viability, while cells in the SDT group had a high death rate (Figure 3(B)). Flow cytometry was used to quantitatively analyze apoptosis (Figure 3(C) and (D)), and the results were consistent with previous results. These results demonstrated that IRO@FA NP-mediated SDT has an excellent antitumor effect in vitro.

### IRO@FA NPs exerted antitumor effects in a mouse model

**Figure 4.**
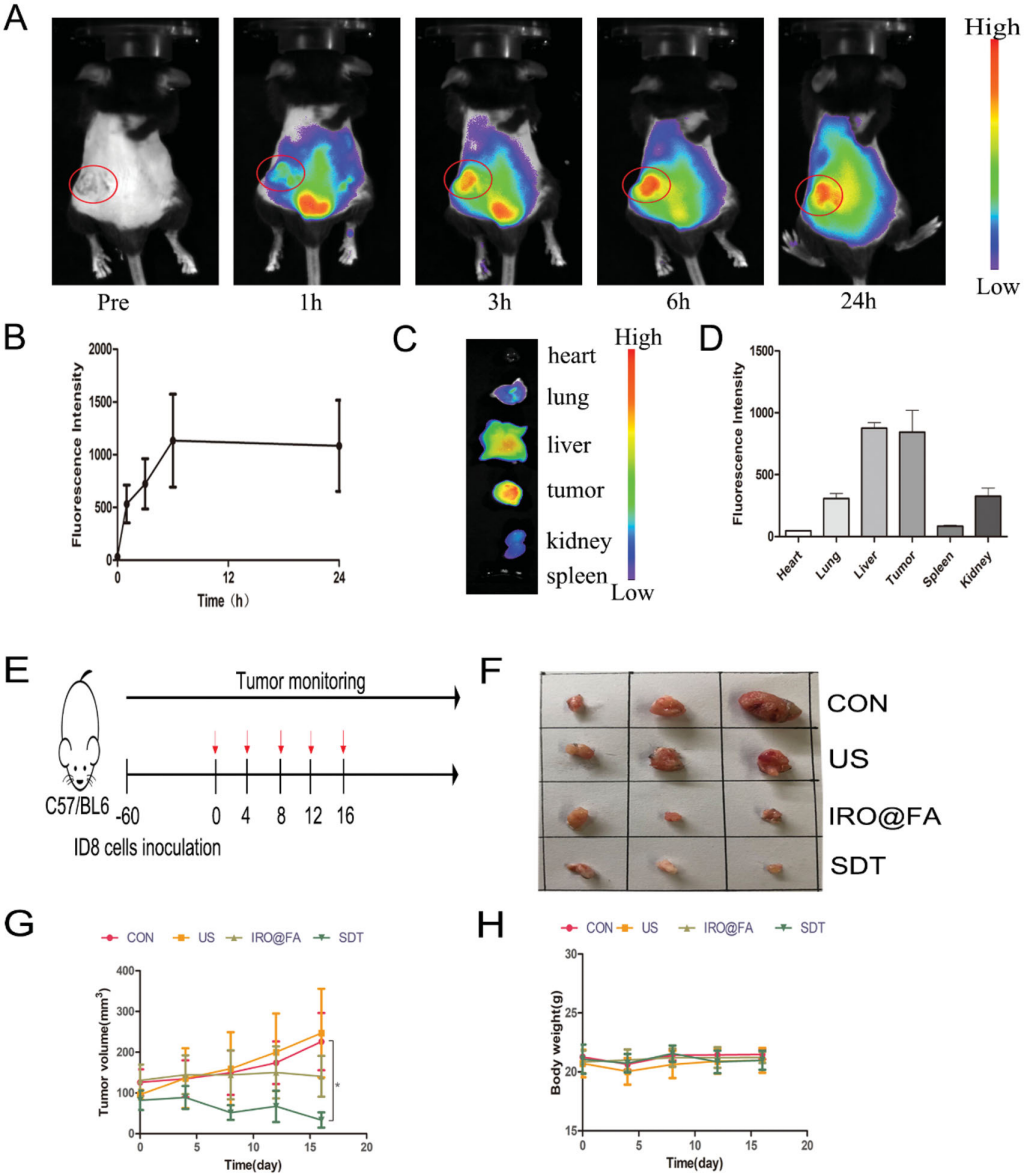
In vivo antitumor effects of IRO@FA NPs. (A) biodistribution of IRO@FA NPs in ID8 tumor-bearing C57/BL6 mice after i.v. injection at different time points (IR780, 1 μg/g); (B) quantification of fluorescence intensity of IR780 from the tumors; (C) distribution of IRO@FA NPs and (D) quantification of fluorescence intensity of IR780 in organs and tumors after injection 24 hours; (E) schematic of therapeutic protocol of mice; (F) photograph of tumors dissected from tumor-bearing mice after different treatments; (G) tumor volume curve and mice weight curve (H) change over time; *p < .05; **p < .01; ***p < .001.

In this study, female C57/BL6 mice with subcutaneous tumors were treated with different conditions when the tumor volume reached more than 30 mm^3^. In vivo fluorescence imaging was used to evaluate the distribution of IRO@FA NPs in the mouse model, and the images were captured at 0, 1, 3, 6, and 24 h. As shown in Figure 4(A), the fluorescence signal accumulated in the tumor area during the first hour, the signal increased over time, and a considerable amount of fluorescence could still be detected after 24 h (Figure 4(B)). Twenty-four hours later, the organs and tumor nodes were isolated for ex vivo fluorescence imaging. Figure 4(C) and (D) indicate that NPs accumulated in tumor tissues and that the signal in tumors was higher than that in other organs, which also provides evidence for follow-up treatment.

The in vivo antitumor effect was further evaluated to explore its potential translation to the clinic. Tumor-bearing mice were randomly divided into four groups: the (I) control group, (II) US only group, (III) IRO@FA NPs alone group, and (IV) SDT group (IRO@FA NPs + US). Two hours post injection, tumors were irradiated by US (2 W, 300 s) every 4 days for 16 days (Figure 4(E)). Two days later, mouse blood, organs and tumor nodes were collected for further analysis. Mouse hearts, livers, kidneys and lungs were fixed for H&E staining (Supplementary Figure S2), and the results indicated good biosafety of the NPs. For the antitumor effects of SDT, the treatment efficacy was directly evaluated by the tumor volume. As shown in Figure 4(F) and (G), the tumor volume in the SDT group increased slowly and was significantly smaller than that in the other groups, and IRO@FA NP-mediated SDT significantly inhibited tumor growth. Moreover, the mouse body weights showed a similar curve (Figure 4(H)) in each group. A TUNEL assay further demonstrated apoptosis occurring in the tumors. As shown in Supplementary Figure S3, tumors in the SDT group had a noticeably higher number of apoptotic cells. These results demonstrated that IRO@FA NPs have good biological safety and that IRO@FA NP-mediated SDT exhibited synergistic therapeutic effects in a mouse model of ovarian cancer.

### IRO@FA NPs induced tumor immune microenvironment variation

**Figure 5.**
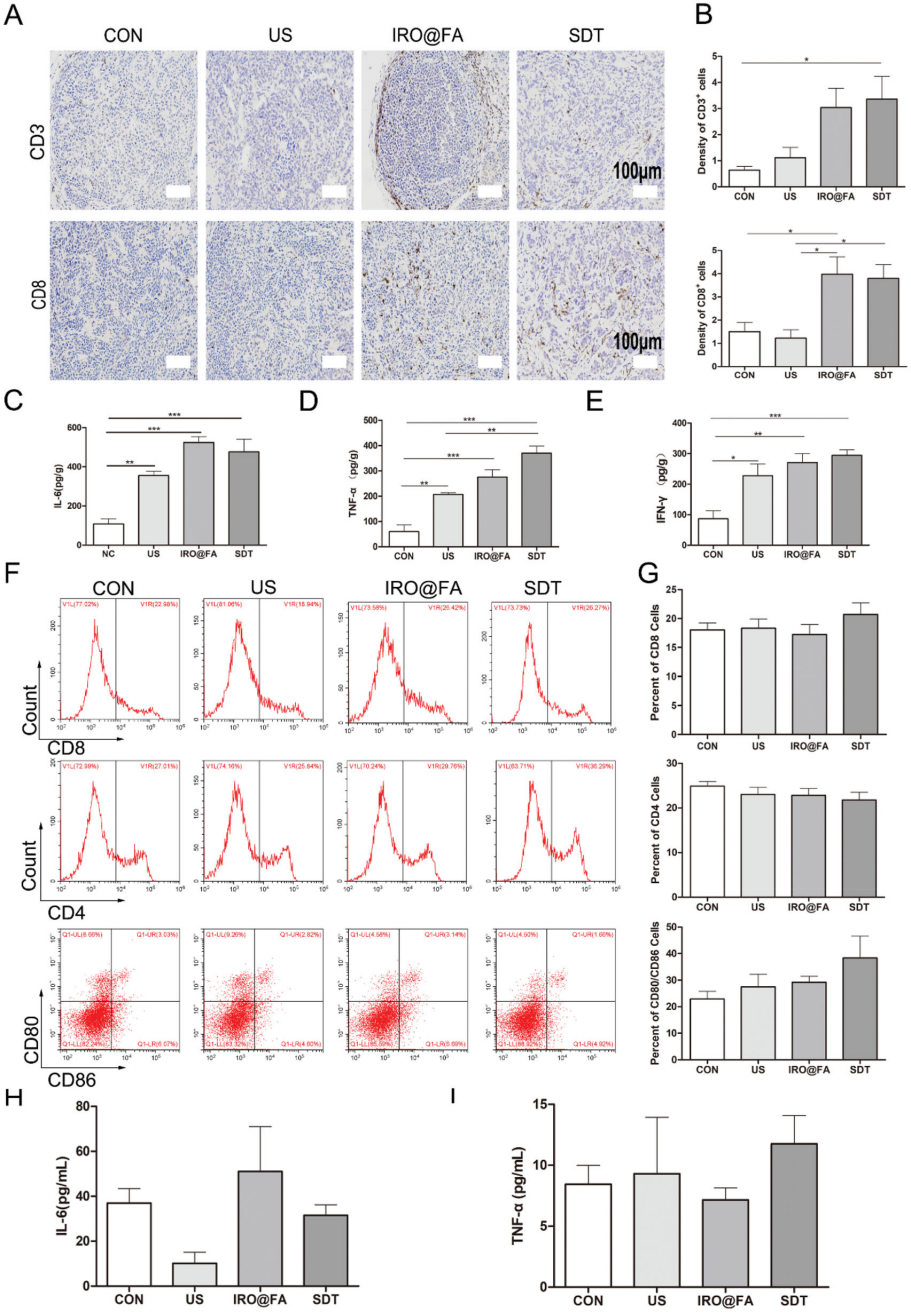
IRO@FA NPs promote intratumoral inflammation and tumor lymphocyte infiltration. (A) immunochemistry staining of CD3^+^ and CD8^+^ cells in tumors; (B) the statistical results of the IOD/Area of CD3^+^ and CD8^+^; (C) intratumoural IL-6, TNF-α (D), IFN-γ (E) determined by ELISA at Day 3 after treatment as above; (F) CD8^+^, CD4^+^and CD80^+^/CD86^+^ cells in mouse spleen and (G) quantification analysis (gated on negative control); (H) IL-6 and (I) TNF-α in blood serum detected by ELISA; *p < .05; **p < .01; ***p < .001.

The density of lymphocyte cells in tumor tissues is positively correlated with the survival rate of ovarian cancer patients. Researchers have also reported that some chemotherapy drugs, such as anthracyclines, are usually associated with immunogenic cell death (ICD) (Obeid et al., 2007) and that increased lymphocyte infiltration enhances antitumor effects (Huang et al., 2019). Inspired by this knowledge, we determined whether IRO@FA NPs triggered by US have the ability to increase tumor lymphocyte infiltration in a mouse model. To answer this question, mice in different groups were euthanized, and their tumor tissues, blood sera, and spleens were collected for further detection. CD3^+^ T and CD8^+^ T cells were labeled with the corresponding antibodies, and their cell densities were measured. Figure 5(A) and (B) show that the densities of CD3^+^ T and CD8^+^ T cells were significantly higher in the SDT group. Additionally, the expression levels of IL-6 (Figure 5(C)), TNF-α (Figure 5(D)) and IFN-γ (Figure 5E) in tumor tissues were detected. The results indicate that the concentrations of these chemokines were higher in mice that received SDT than in the PBS-treated group.

Lymphocyte cells were extracted from the mouse spleens and labeled with CD80/CD86, CD8 and CD4, and then flow cytometry was utilized to count the positive cells isolated from the spleens. The results indicated that the proportions of CD8^+^ and CD80^+^CD86^+^ cells were higher in the SDT group than in the other groups, but the difference was not significant (Figure 5(F) and (G)). Moreover, the expression levels of IFN-γ, IL-6 and TNF-α in blood serum were detected, and the concentrations of IL-6 and TNF-α were not significantly different among the groups, while the concentration of IFN-γ was too low to be detected (Figure 5(H) and (I)). In brief, IRO@FA NP-mediated SDT induced an increase in tumor-infiltrated lymphocytes and an upregulation of inflammatory markers. These results demonstrated that SDT induced inflammation in the tumor niche and modulated the tumor immune microenvironment.

### IRO@FA NPs induced ID8 cell ICD and DC activation *in vitro*

Cells undergoing injury, stress and death may synthesize and secrete damage-associated molecular patterns (DAMPs), such as high mobility group protein 1 (HMGB1), ATP and eversion of calreticulin (CRT). DAMPs are recognized by pattern recognition receptors (PRRs) on DCs, promote the maturation of DCs and activate the immune response (Galluzzi et al., 2017). This kind of death occurring in tumor cells is commonly referred to as immunogenic cell death (ICD) (Kepp et al., 2014; Galluzzi et al., 2020). Tumor cells that experience ICD can be used as vaccines and act as immune adjuvants that activate an adaptive immune response in immunocompetent syngeneic hosts.

**Figure 6.**
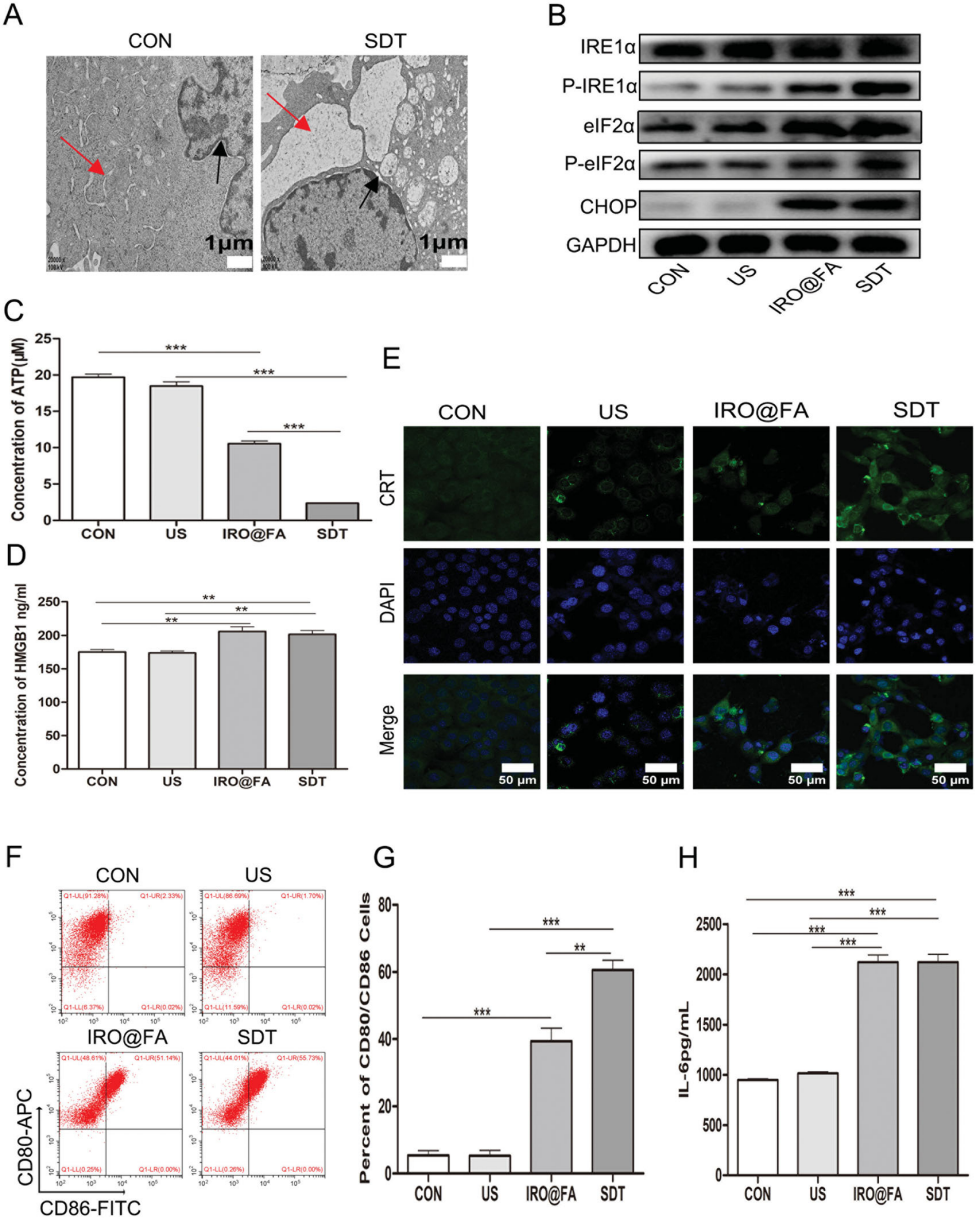
IRO@FA NPs induced secretion of DAMPs and prompted DCs activation in vitro. (A) after exposure to SDT, cells were processed for TEM, the morphology of ER was observed. The red arrow represented ER. The black arrow represent perinuclear space; (B) after treated with NPs and SDT for 24 hours, cells were subjected to western blot analyzing using the indicated antibodies, GAPDH was used as loading control; (C) cells were treated as above and retained amount of ATP was determined; (D) the concentration of HMGB1 in cell supernatants were determined with ELISA; (E) calreticulin on cell membrane were detected with immunofluorescence and observed by LSCM; (F) DCs from C57/BL6 mice were cultured in vitro and incubated with different cell supernatants, proportion of activated DCs were counted by flow cytometry (gated on negative control); (G) showed quantification analysis of (F); (H) the concentration of IL-6 in supernatants from DCs treated with different conditions were analyzed by ELISA; *p < .05; **p < .01; ***p < .001.

ROS and other stimuli lead to an increase and imbalance in the load of unfolded proteins, which disturbs ER homeostasis and induces ER stress. ER stress is an important intracellular pathway that governs ICD (Krysko et al., 2012). Based on these factors, we determined whether IRO@FA NP-mediated SDT induced ER stress and ICD in ID8 cells. Transmission electron microscopy was used to observe exact cell morphology changes. Figure 6(A) shows ER edema and an increase in the perinuclear space in the cells of the SDT group. Furthermore, the ER stress-associated pathways eIF2α and IRE1α were activated in the cells of the IRO@FA and SDT groups, as indicated by protein phosphorylation and overexpression (Figure 6(B)). Moreover, the lower ATP content retained in the SDT group cells means that more ATP was released into the supernatant (Figure 6(C)). Figure 6(D) shows that HMGB1 was significantly increased in the cells of the SDT group. Furthermore, the LSCM images showed increased expression and membrane relocation of CRT in cells in the IRO@FA and SDT groups (Figure 6(E)). These results suggest that ER stress and cell function changes occurred in cells in the SDT group and that IRO@FA NP-mediated SDT triggered ER stress and DAMP secretion.

To investigate whether SDT-treated cells could activate an immune response in vitro, DCs were incubated with the supernatant from ID8 cells for 24 hours. Then, the cells were collected and labeled with CD80/CD86, and double-positive cells were detected and counted with flow cytometry. Figure 6(F) and (G) show that the proportion of CD80^+^CD86^+^ cells was higher in the SDT group than in the control group. Moreover, ELISA was utilized to test the expression level of IL-6 in the DC supernatant, and consistent with the previous results, the expression level of IL-6 was significantly higher in the SDT group than in the others (Figure 6(H)). These results suggest that DCs were activated after stimulation with the ID8 cell supernatant and indicate that SDT induced ID8 cell ICD and initiated the immune response.

### Increased expression of DAMPs in a mouse model

**Figure 7.**
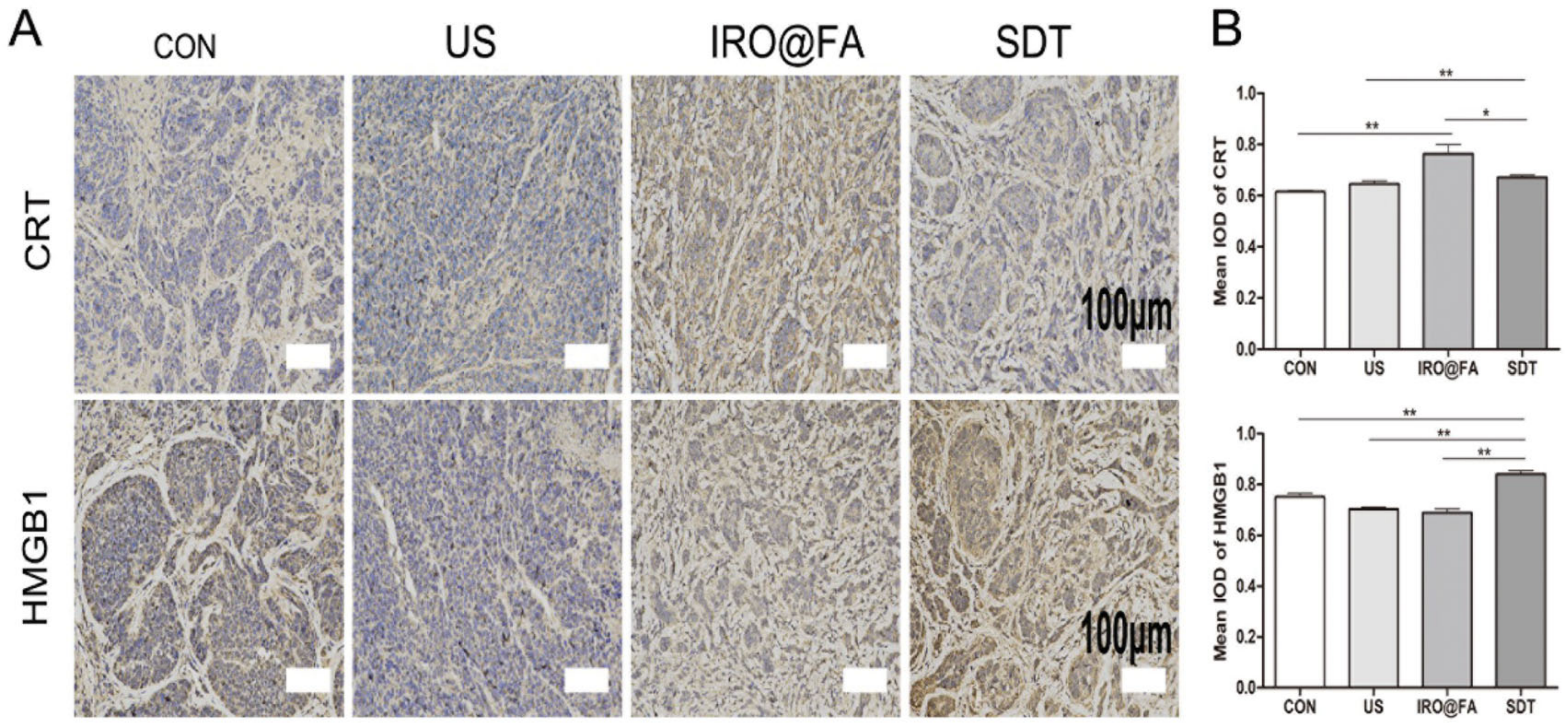
Increased expression of DAMPs in tumors. (A) CRT and HMGB1 expression in tumors were detected by immunochemistry; (B) the statistical results of the IOD/Area of Calreticulin and HMGB1; *p < .05; **p < .01; ***p < .001.

The results of our in vitro studies prompted us to investigate whether ICD also occurs in a mouse model. We analyzed protein expression in tumor samples from tumor-bearing mice treated with different conditions. The expression of CRT and HMGB1 was significantly higher in the SDT group (Figure 7(A) and (B)), and the results demonstrated that IRO@FA-mediated SDT has the ability to induce ICD in vivo.

## Discussion

EOC has the highest mortality rate among gynecologic malignancies (Kuroki & Guntupalli, 2020). The majority of patients enter remission after cytoreductive combined platinum-based chemotherapy; however, more than 50% of these patients experience tumor recurrence and death within five years (Fucikova et al., 2021). Immune checkpoint inhibitors (ICIs) are among the most promising methods for cancer treatment, but based on several clinical trials, their effect in ovarian cancer is not good, and data from the pembrolizumab standalone clinical trial Keynote-100 showed that the ORR was 8% in PD-L1-negative patients and 18% in PD-L1-positive patients (Matulonis et al., 2019). A possible reason for this may be due to indolent anticancer immunity and robust baseline immunosuppressive conditions (Galon & Bruni, 2019). Few patients can benefit from single-agent ICIs, and the implementation of combined strategies is urgently needed.

Photothermal therapy and sonodynamic therapy have been demonstrated to boost lymphocyte infiltration and augment the antitumor effects of ICIs (Liang et al., 2019; Zhang et al., 2020; Si et al., 2021) reported that pure oxygen-carrying NPs triggered by US also have the ability to activate antitumor immunity in the body. Combining nanomedicine with immunotherapy is an important method to improve therapeutic effects (Ye et al., 2019; Lin et al., 2021). Our study showed that IRO@FA NP-mediated SDT induced the infiltration of CD3^+^ T and CD8^+^ T cells. Moreover, the expression levels of the inflammatory chemokines IL-6, TNF-α and IFN-γ were upregulated intratumorally. Additionally, the expression level of PD-L1 in tumor sections was significantly upregulated after treatment with IRO@FA NPs or SDT (Supplementary Figure S4). High expression of PD-L1 may contribute to tumor evasion, but it can also act as a target for anti-PD-L1 therapy. Our study indicated that SDT modulated the tumor immune microenvironment and promoted inflammation in the tumor milieu, which suggested that SDT has multiple antitumor effects and can be a potential adjuvant to ICI therapy.

The tumor microenvironment comprises a heterogeneous mixture of different lymphocytes, fibroblasts, neurons and endothelial cells, and their compositions and spatial distributions vary across cancer types. The presence of tumor-infiltrating lymphocytes in EOC is positively correlated with patient overall survival, especially in ovarian cancer (Hwang et al., 2012), and improving tumor lymphocyte infiltration is a key approach to improve therapeutic effects. However, strategies for turning “cold” tumors into “warm” or “hot” have faced hurdles (van der Woude et al., 2017), including abnormalities in tumor vessels, deficiencies of antigen-presenting cells, a lack of tumor antigens and a tumor suppressive environment (Bonaventura et al., 2019). Inducing tumor cell ICD is an important method to release tumor-associated antigens and promote antigen-presenting cell activation (Galon & Bruni, 2019). In view of the decisive role of ER stress in ICD (Chen & Cubillos-Ruiz, 2021), many studies have aimed to target the ER and induce ICD (Deng et al., 2020). In this study, SDT triggered ER stress that elicited downstream DAMP/danger signaling pathways, amplified ICD and activated immune cells. DAMPs are important immune adjuvants for the maturation of antigen-presenting cells: CRT is evenly distributed in the cytoplasm of resting cells; once activated, it translocated to the outer membrane leaflet, which enables phagocytes and efficient engulfment of dead cells (Kasikova et al., 2019). Extracellular secreted HMGB1 induces severe inflammation through TLR2 and TLR4 and facilitates antigen processing and cross presentation through combination with TLR4 on DCs (Chiba et al., 2012). Extracellular ATP is also known as a “find me” signal for apoptotic cells, promoting antigen-presenting cells to find them and to activate the immune system (Michaud et al., 2011). Our in vitro immune activation assay demonstrated that cell supernatants from the SDT group upregulated the expression of DAMPs and had the ability to induce an immune response in antigen-presenting cells. These results indicated that SDT is a potential method to turn “cold” tumors into “hot” tumors and may improve the therapeutic effects of ICIs. Anthracyclines have been reported to induce tumor cell ICD (Shu et al., 2020); however, a previous clinical trial demonstrated that patients who accepted ICIs combined with PEGylated liposomal doxorubicin did not show improvements in PFS and OS (Pujade-Lauraine et al., 2021), and the real effects of SDT still need to be explored.

Based on the mechanisms used to induce ER stress, ICD inducers are divided into two categories: Type I inducers, such as mitoxantrone and cyclophosphamide, target cytosolic proteins or DNA-replication proteins rather than targeting the ER directly and induce ER stress through secondary or collateral effects; Type II inducers selectively target the ER and disturb ER homeostasis. Moreover, the coexistence of ROS and ER stress can significantly increase the secretion of DAMPs (Krysko et al., 2012; Wang et al., 2021), which is why ICD was more significant in the SDT group. However, in our study, ER stress occurred not only in the SDT group but also in the IRO@FA group, and IRO@FA NPs alone also induced ICD. The mechanism of IRO@FA NP-induced ER stress and ICD is unclear. Zhang et al. reported that IR780 can selectively target mitochondria (Wang et al., 2020), and colocalization of IR780 and mitochondria may influence mitochondrial membrane potential, leading to mitochondrial dysfunction (Zhang et al., 2021). Mitochondria and ER are both membrane-bound organelles and form physical contacts in specific domains (Hayashi et al., 2009). Whether mitochondrial dysfunction can induce ER stress and how it occurs need to be investigated.

## Conclusion

In this study, we successfully constructed IRO@FA NPs with a spherical morphology and an appropriate diameter. In an in vitro study, IRO@FA NPs irradiated with US produced sufficient ROS and showed good targeting ability and toxicity to tumor cells at a certain concentration. In an in vivo study, IRO@FA NPs displayed good biosafety in mice, and subcutaneous tumor growth was obviously inhibited. We also observed local immune activation and increased lymphocyte infiltration of the tumor microenvironment in both the IRO@FA group and the SDT group. To explore the underlying mechanisms of the immune response, we performed additional studies and found that ICD was involved in the death of IRO@FA NP- treated cells.

**Scheme 1.**
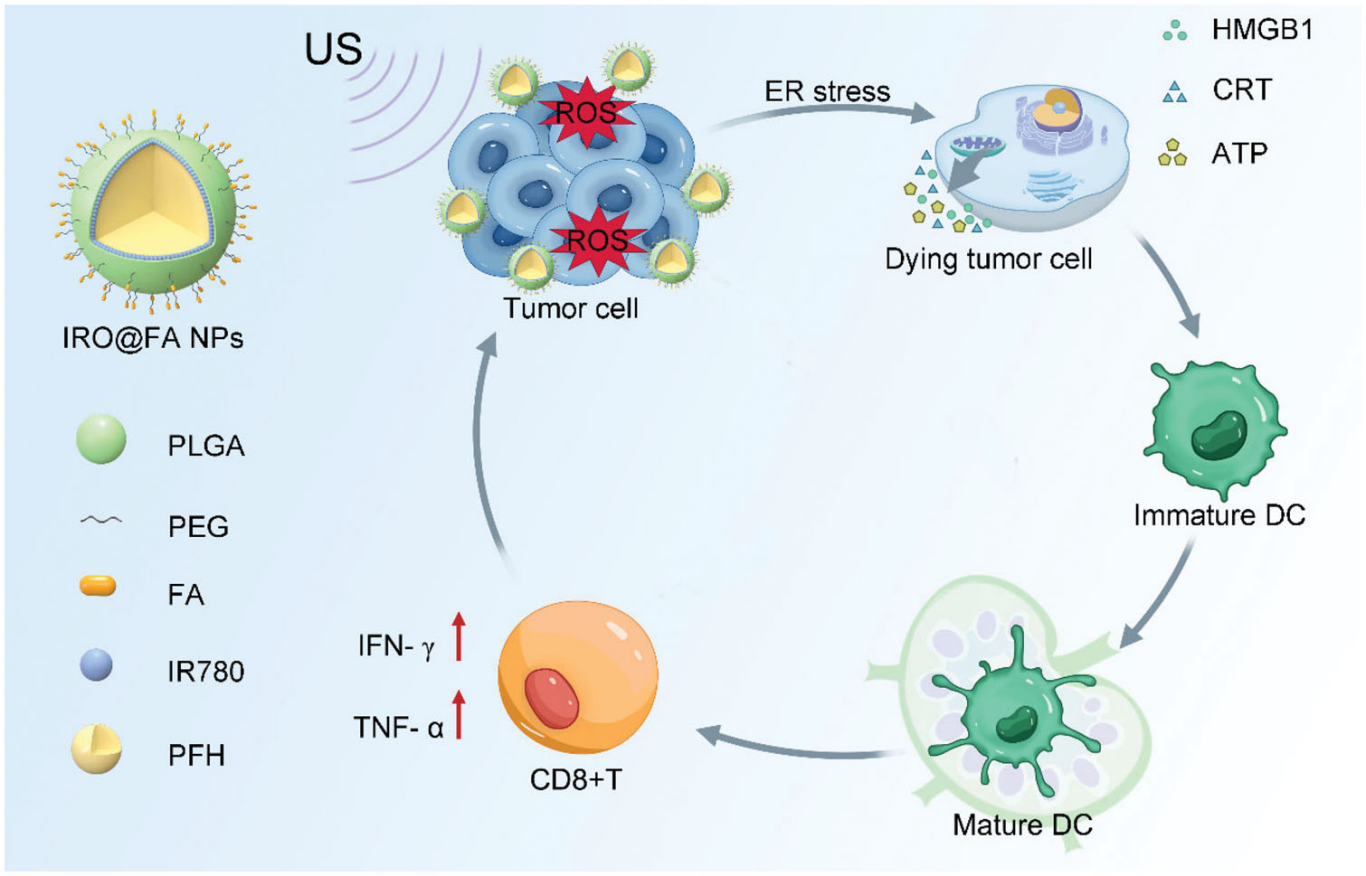
The composition of IRO@FA NPs and schematic illustration of the synergistic therapy.

## Supplementary Material

Supplemental Material
