# Supplementary material for: Sonosensitizer nanoplatform-mediated sonodynamic therapy induced immunogenic cell death and tumor immune microenvironment variation
Source: Drug Deliv. 2022 Apr 8;29(1):1164–75. doi: 10.1080/10717544.2022.2058653 (PMC9004507; doi:10.1080/10717544.2022.2058653)
Supplement: Supplemental Material [file IDRD_A_2058653_SM3769.docx]

**Supplementary data**


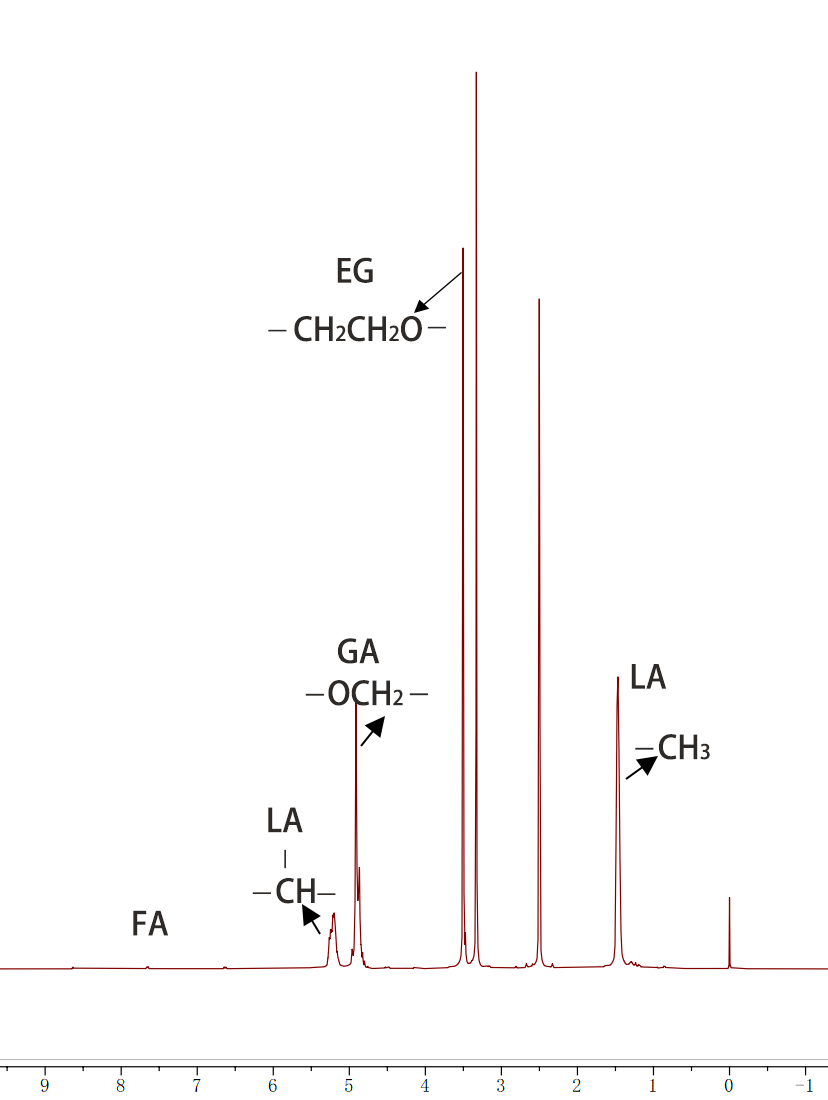


**Figure S1** ^1^H NMR (600MHz) spectrum of PLGA-PEG-FA.


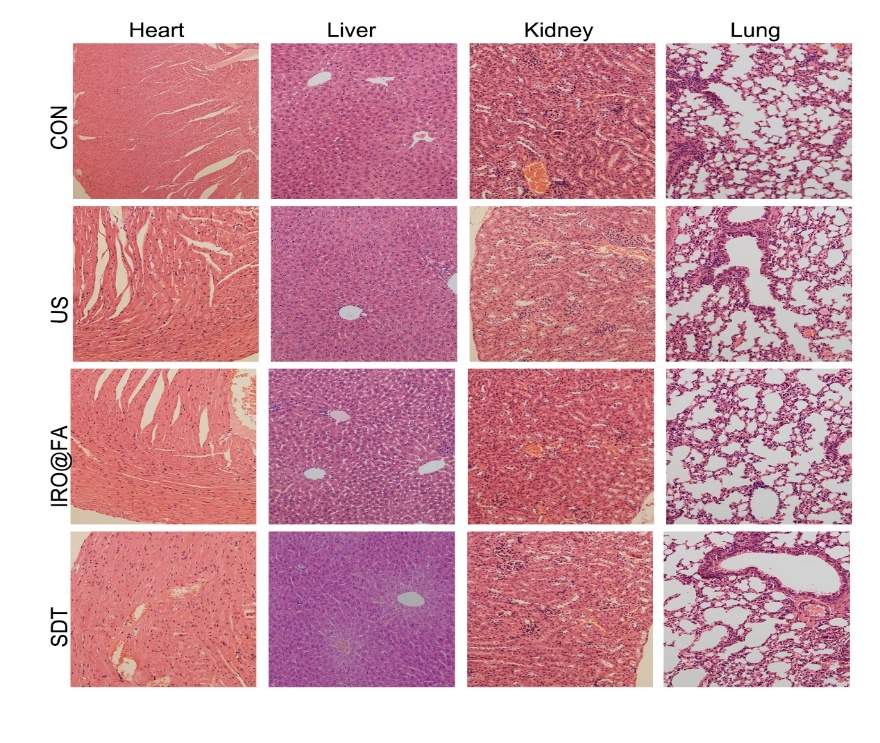


**Figure S2** H&E staining of mice heart, liver, kidney and lung after treated by PBS, US, IRO@FA NPs and SDT (IRO@FA NPs plus US).


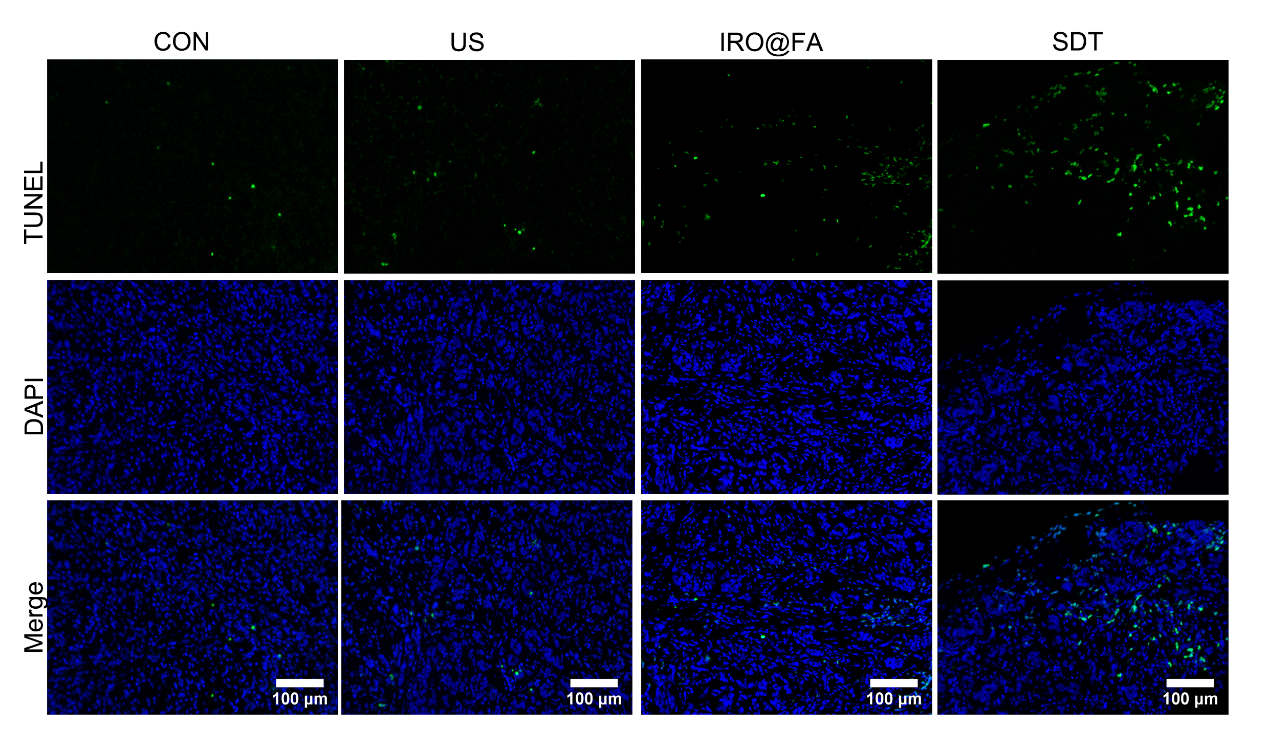


**Figure S3** TUNEL assay of tumor sections for different groups.


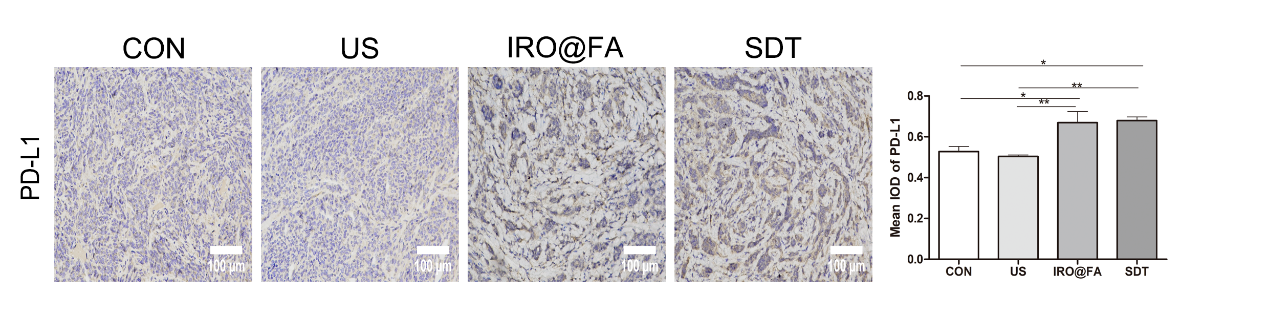


**Figure S4** Immunochemistry staining of PD-L1^+^ cells in tumors and quantification analysis.


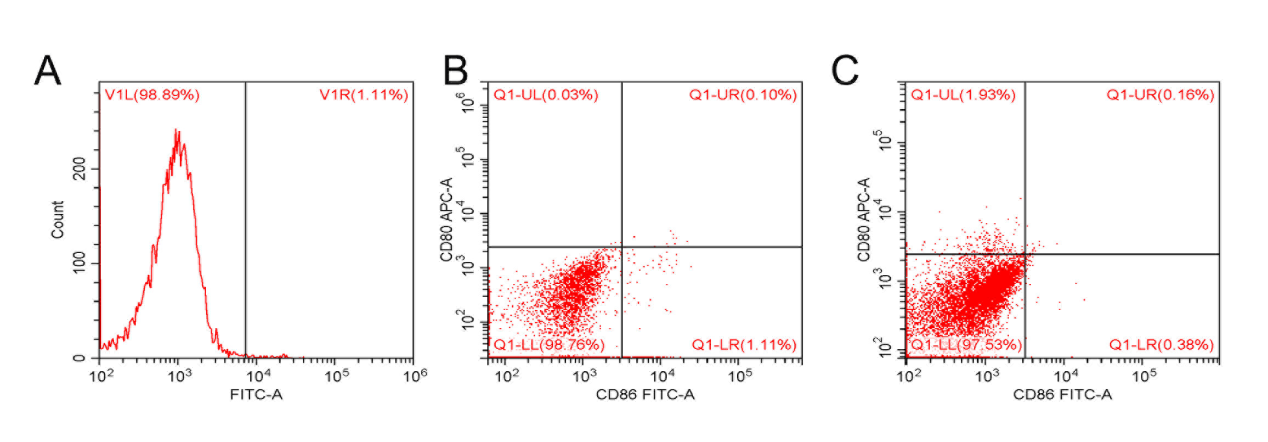


**Figure S5** negative control of flowcytometry. (A) negative control for CD4 and CD8 cells in vivo; (B) negative control for CD80/CD86 cells in vivo; (C) negative control for CD80/CD86 cells in vitro.
